# Absence of 4-1BB reduces obesity-induced atrophic response in skeletal muscle

**DOI:** 10.1186/s12950-017-0156-5

**Published:** 2017-05-11

**Authors:** Ngoc Hoan Le, Chu-Sook Kim, Thai Hien Tu, Byung-Sam Kim, Taesun Park, Jung Han Yoon Park, Tsuyoshi Goto, Teruo Kawada, Tae Youl Ha, Rina Yu

**Affiliations:** 1 Department of Food Science and Nutrition, University of Ulsan, Ulsan, 44610 South Korea; 2 Department of Biological Science, University of Ulsan, Ulsan, 44610 South Korea; 3 Department of Food and Nutrition, Yonsei University, Seoul, 03722 South Korea; 4 Department of Food Science and Nutrition and Research Institute for Bioscience & Biotechnology, Hallym University, Chuncheon, 24252 South Korea; 5 Graduate School of Agriculture, Kyoto University, Uji, Kyoto 611-0011 Japan; 6 Research Group of Nutrition and Metabolic System, Korea Food Research Institute, Seongnam, 13539 South Korea

**Keywords:** Obesity, Inflammation, Oxidative metabolism

## Abstract

Obesity-induced inflammation causes skeletal muscle atrophy accompanied by disruption of oxidative metabolism and is implicated in metabolic complications such as insulin resistance and type 2 diabetes. We previously reported that 4-1BB, a member of the tumor necrosis factor receptor superfamily, participated in obesity-induced skeletal muscle inflammation. Here, we show that the absence of 4-1BB in obese mice fed a high-fat diet led to a decrease in expression of atrophic factors (MuRF1 and Atrogin-1) with suppression of NF-κB activity, and that this was accompanied by increases in mitochondrial oxidative metabolic genes/proteins (e.g., PGC-1α, CPT1β, etc.) expression and oxidative muscle fibers marker genes/proteins in the skeletal muscle. These findings suggest that 4-1BB-mediated inflammatory signaling could be a potential target for combating obesity-related muscle atrophy and metabolic derangement in skeletal muscle.

## Background

Skeletal muscle, which constitutes up to 40% of body mass, plays a pivotal role in energy metabolism, protein storage, and locomotion. Thus, the maintenance of skeletal muscle mass and function are important for preventing obesity and metabolic complications such as insulin resistance and type 2 diabetes. Growing evidence suggests that obesity induces sarcopenia, a loss of skeletal muscle mass referred to as atrophy or wasting, resulting in increased risk of metabolic complications [1, 2]. Obesity-induced skeletal muscle inflammation is a major contributor of the skeletal muscle loss/atrophy. The skeletal muscle inflammation is characterized by increases in intramuscular adipocytes, recruitment of macrophages, and various inflammatory cytokines such as tumor necrosis factor alpha (TNFα), interleukin-6 (IL-6), and monocyte chemoattractant protein-1 (MCP-1) [3, 4]. Although inflammatory cytokines are considered to enhance muscle catabolism and play a major role in the development of skeletal muscle loss/atrophy [4, 5], molecules and cellular mechanisms involved in the obesity-induced muscle atrophy are not yet fully understood.

Skeletal muscle atrophy occurs through an imbalance between protein synthesis and degradation, leading to a decrease in the size of muscle. Two major proteolytic systems involved in muscle atrophy are the ubiquitin-proteasome pathway and the autophagy-lysosome pathway [6]. Transcription factors such as nuclear factor-kappa B (NF-κB) and Forkhead box O (FoxO3) are key mediators of the catabolic response during muscle atrophy in various physiological and pathophysiological conditions, including obesity [7]. Obesity-induced skeletal muscle inflammation leads to degradation of specific muscle proteins and blockade of the regeneration of myofibers by activation of NF-κB [8]. Recent studies have shown that the inflammatory receptor fibroblast growth factor inducible 14 (Fn14) modulates skeletal muscle atrophy and metabolism [9, 10] through binding to its ligand tumor necrosis factor-like weak inducer of apoptosis (TWEAK). The interaction of Fn14/TWEAK increases activation of NF-κB signaling and proteolytic pathways such as the ubiquitin-proteasome system [9], and this is accompanied by reduction of mitochondrial oxidative metabolism and fiber type switching in skeletal muscle [11, 12], indicating that the inflammatory receptor/ligand system participates in obesity-induced muscle atrophy and metabolic dysregulation as well.

4-1BB (TNF superfamily 9, TNFRSF9) is an inflammatory receptor expressed on the surface of both immune cells (e.g., T cells) and non-immune cells (e.g., endothelial cells, adipocytes, and myotubes) [13–16]. 4-1BB signaling regulates various inflammatory processes by modulating cytokine production and cell proliferation/survival [17–19]. We previously showed that 4-1BB deficiency attenuates obesity-induced adipose inflammation and metabolic complications such as insulin resistance in mice fed a high-fat diet (HFD) [16], and that in vitro stimulation of 4-1BB promoted free fatty acid-induced inflammatory response in muscle cells through NF-κB activation [3]. In this study, we tested the hypothesis that absence of 4-1BB-mediated signaling reduces obesity-induced skeletal muscle atrophy and metabolic dysregulation in mice fed an HFD.

Here, we demonstrate that the absence of a 4-1BB-mediated signal reduces obesity-induced atrophic responses in skeletal muscle by suppressing NF-κB activation, and that this is associated with increased activation of adenosine monophosphate-activated protein kinase (AMPK) and mitochondrial oxidative metabolism accompanied by increased oxidative fiber type in the skeletal muscle. These findings suggest that disruption of the 4-1BB signal may protect obesity-induced sarcopenia and metabolic dysregulation.

## Methods

### Animals

The whole-body 4-1BB-deficient mice on a C57BL/6 background were established in the Immunomodulation Research Center of University of Ulsan [20]. Male 4-1BB-deficient mice and their wild type (WT) littermate at 8 weeks of age were individually housed in plastic cages in a specific pathogen-free animal facility with a 12-h light, 12-h dark cycle. The mice were fed a high-fat diet (HFD) (60% of calories from fat; #D12492; Research Diets, New Brunswick, NJ, USA) or a regular diet (RD) (13% of calories from fat; #2018; Harlan Teklad, Madison, WI, USA) for 9 weeks, and given free access to food and water. All animal care and procedures were conducted according to the protocols and guidelines approved by the University of Ulsan Animal Care and Use Committee (2011-HA-004). Mice were euthanized by CO_2_ asphyxiation and skeletal muscles were dissected.

### Quantitative real-time PCR (qRT-PCR)

Quadriceps muscle tissues were collected and stored at −20 °C in RNAlater (Ambion, Austin, TX, USA). Total RNA was extracted from 50 mg muscle tissue samples with Tri-reagent (Life Technologies, Carlsbad, CA, USA). Two microgram aliquots of total RNA were reverse transcribed to cDNA using M-MLV reverse transcriptase (Promega, Madison, WI, USA). qRT-PCR amplification of the cDNA was performed using SYBR premix Ex Taq (TaKaRa Bio Inc, Forster, CA, USA) in a Thermal Cycler Dice (TaKaRa Bio Inc, Japan). All reactions were performed using the following schedule: 95 °C for 10s and 45 cycles of (95 °C for 5 s and 60 °C for 30s). Results were analyzed with the Real Time System TP800 software and all values were normalized to the levels of the house-keeping gene, β-actin [21, 22], which was unaffected by genotype (WT: 1.00 ± 0.10, KO: 1.25 ± 0.08, *p* < 0.0675). The primers used in the analysis are listed in Table 1.

**Table 1:** Mouse primers used for qRT-PCR

Gene	Forward primer (5` → 3`)	Reverse primer (5` → 3`)
MuRF1	TGTCTCACGTGTGAGGTGCCTA	CACCAGCATGGAGATGCAGTTAC
Atrogin-1	ACATTCTGCCAGCTGCTGTTTC	TGAGTTGGATGCTGGGCCTAC
PGC-1α	CCGTAAATCTGCGGGATGATG	CAGTTTCGTTCGACCTGCGTAA
COX8B	AAAGCCCATGTCTCTGCCAAG	TGGAACCATGAAGCCAACGA
NDUFA2	ATCGTGCAACGGTACGTGGA	CCTTCAGGCTTTGCCGCTTA
PPARα	ACGCTCCCGACCCATCTTTAG	TCCATAAATCGGCACCAGGAA
CPT1β	GAGACAGGACACTGTGTGGGTGA	AGTGCCTTGGCTACTTGGTACGAG
ACADM	TGATGTGGCGGCCATTAAGA	GGGTTAGAACGTGCCAACAAGAA
ACADL	CATCCTCATGCAAGAGCTTCCACA	CTTCAAACATGAACTCACAGGCAGA
MYL2	AACAGAGACGGCTTCATCGACA	CGGTGAAGTTAATTGGACCTGGA
MYH7	GCGCAATGCAGAGTCAGTGAA	TGCAGCTTGTCCACCAGGTC
myoglobin	TGGCAGCTGGTGCTGAATG	CCAGGGTCTCAGGGTGAGTCTTA
TNNI1	TCACCTGCACAGGACACGAAC	CCTTGGCCTTGGCTAGCATC
TNNT1	TCAATGTGCTCTACAACCGCATC	GTCATGTCCTGGCAGTCTCACTTC
MYHC2	TTCTCAGGCTTCAGGATTTGGTG	CTTGCGGAACTTGGATAGATTTGTG
TNNI2	CGAAGATCGACGTGGCTGAA	CATGGCGTCGGCAGACATAC
TNNT3	CATGGGTGCCAACTACAGCAG	CAGCTTGTCATCGCTAAGATGGTC
TNNC2	ACGGCCGCATTGACTTTGA	CTGCAGTCTGGATGGACACGA
MYHC4	GCTGCAGGACTTGGTGGACA	GGCCAGGTTGACATTGGATTG
β-actin	CATCCGTAAAGACCTCTATGCCAAC	ATGGAGCCACCGATCCACA

*MuRF1* muscle RING finger 1, *PGC-1α* peroxisome proliferator-activated receptor gamma coactivator-1 alpha, *COX8B* cytochrome c oxidase, subunit VIIIB, *NDUFA2* NADH dehydrogenase ubiquinone 1 alpha subcomplex 2, *PPARα* peroxisome proliferator activated receptor alpha, *CPT1β* carnitine palmitoyltransferase 1 beta, *ACADM* acyl coenzyme A dehydrogenase, medium chain, *ACADL* acyl coenzyme A dehydrogenase, long-chain, *MYL2* myosin, light chain 2, *MYH7* myosin, heavy chain 7, *TNNI1* troponin I, skeletal, slow 1, *TNNT1* troponin T, skeletal, slow 1, *MYHC2* myosin heavy chain 2, *TNNI2* troponin I type 2, *TNNT3* troponin T type 3, *TNNC2* troponin C type 2, *MYHC4* myosin heavy chain 4

### Western blot analysis

Mice were euthanized by CO_2_ asphyxiation. Briefly, frozen whole quadriceps muscle tissues were homogenized in lysis buffer containing 150 mM NaCl, 50 mM Tris–HCl, 1 mM EDTA, 50 mM NaF, 10 mM Na_4_P_2_O_7_, 1% IGEPAL, 2 mM Na_3_VO_4_, 0.25% protease inhibitor cocktail, and 1% phosphatase inhibitor cocktail (Sigma). The protein content was determined using a BCA Protein Assay Kit (Pierce, Rockford, IL, USA). Proteins were resolved on 11% SDS-PAGE gels, transferred onto nitrocellulose membranes (Millipore, Tullagreen, Carrigtwohill, Ireland, USA), and immunoblotted using primary antibodies against the following proteins: anti-phospho AMPK (Thr172, #2531), AMPK (#2332, Cell Signaling, Beverly, MA, USA), COX IV (ab33985, Abcam, Cambridge, MA, USA) MuRF1 (#sc-32920, Santa-Cruz Biotechnology, CA, USA), PGC-1α (ab54481, Abcam), Troponin I (slow) (#sc-8119, Santa-Cruz) and α-tubulin (ab7291, Abcam). Proteins sequentially were detected by anti-rabbit (Cell Signaling) or anti-mouse (Bethyl Laboratories, Montgomery, TX, USA) HRP-conjugated secondary antibody, and enhanced chemiluminescent substrate kit (PerkinElmer, Waltham, MA, USA). Intensity of protein bands was quantified by densitometry using Image J program.

### Nuclear factor-κB activity

Nuclear factor-κB (NF-κB) DNA binding activity was assessed with a NF-κB p65 TransAM kit (Active Motif, Rixensart, Belgium). Samples of tissue homogenate normalized for protein content were incubated with immobilized oligonucleotides containing an NF-κB consensus binding site. DNA binding activity was analyzed with antibodies specific for the NF-κB subunits according to the manufacturer’s instructions (Active Motif).

### Immunohistological analysis

Quadriceps muscles were fixed overnight at room temperature in 10% formaldehyde, after which they were embedded in paraffin. Next, 8 μm thick sections were stained with anti-COX IV (Abcam) and anti-Troponin I (slow) (Santa-Cruz). Secondary antibodies were goat anti-mouse (Southern Biotechnology Associates, Inc., Birmingham, AL, USA), and donkey anti-goat (Santa Cruz Biotechnology), respectively, and detection was accomplished using a Peroxidase Substrate kit (Vector Laboratories Inc., Burlingame, CA, USA).

### Statistical analysis

The results are presented as the means ± standard error of the mean (SEM). Differences were analyzed with the Student’s *t* test, and they were considered significant at *P* < 0.05.

## Results

### Muscle weight, atrophic response, and NF-κB activation in skeletal muscle

**Fig. 1:**
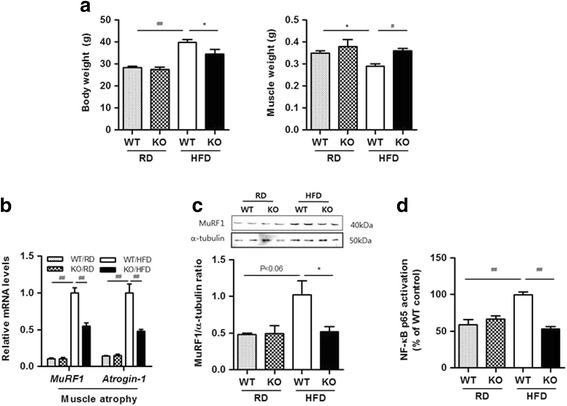
Effect of 4-1BB deficiency on muscle weight, atrophic response, and NF-κB activation **a** Body weights and quadriceps muscle weights. **b** qRT-PCR analysis for markers specific to atrophic factors (MuRF1 and Atrogin-1) in quadriceps muscles. **c** MuRF1 and a-tubulin protein were measured by Western blotting. The relative intensities of the bands of MuRF1/α-tubulin were measured using image J. **d** NF-κB activation in quadriceps muscles was determined using the p65 TransAM assay as described in the Materials and Methods. Data are the means ± SEM; n = 4–6 in each group. *P < 0.05, # *P* < 0.005, #*# P* < 0.001 compared with WT/HFD group

Obesity-induced inflammation is closely associated with skeletal muscle atrophy, which occurs through enhanced protein degradation and/or reduced protein synthesis [23, 24]. We previously reported that 4-1BB provides an inflammatory signal in muscle cells, and that disruption of the interaction between 4-1BB and its ligand decreased the production of inflammatory cytokines from cocultured muscle cells/macrophages [25]. In this study, we examined whether absence of 4-1BB represses obesity-induced atrophic response in skeletal muscle. We first observed that body weights of the 4-1BB–deficient mice given an HFD were significantly lower than those of the HFD-fed WT mice, whereas there was no difference between WT and 4-1BB–deficient mice fed a RD (Fig. 1a). The weights of the quadriceps muscle tissue were lower in the HFD-fed than in the RD-fed mice, and this reduction was prevented in the HFD-fed 4-1BB-deficient mice (Fig. 1a). Next, we determined atrophic response in the skeletal muscle of WT and 4-1BB-deficent mice fed a RD or HFD. As shown in Fig. 1b, transcript levels of atrophic genes such as MuRF1 and Atrogin-1 were upregulated in the HFD-fed mice, and were reduced in the skeletal muscle of the HFD-fed 4-1BB-deficient mice than in that of the HFD-fed WT mice. Western blot analysis revealed that the levels of atrophic protein such as MuRF1 were downregulated in the HFD-fed 4-1BB-deficient mice than in the HFD-fed WT mice (Fig. 1c). Subsequently, we confirmed that the inflammatory signaling estimated by activity of the NF-κB subunit p65 is suppressed in the skeletal muscle of the HFD-fed 4-1BB-deficient mice and is similar to the level in the RD-fed mice (Fig. 1d).

#### Mitochondrial oxidative gene expression in skeletal muscle

**Fig. 2:**
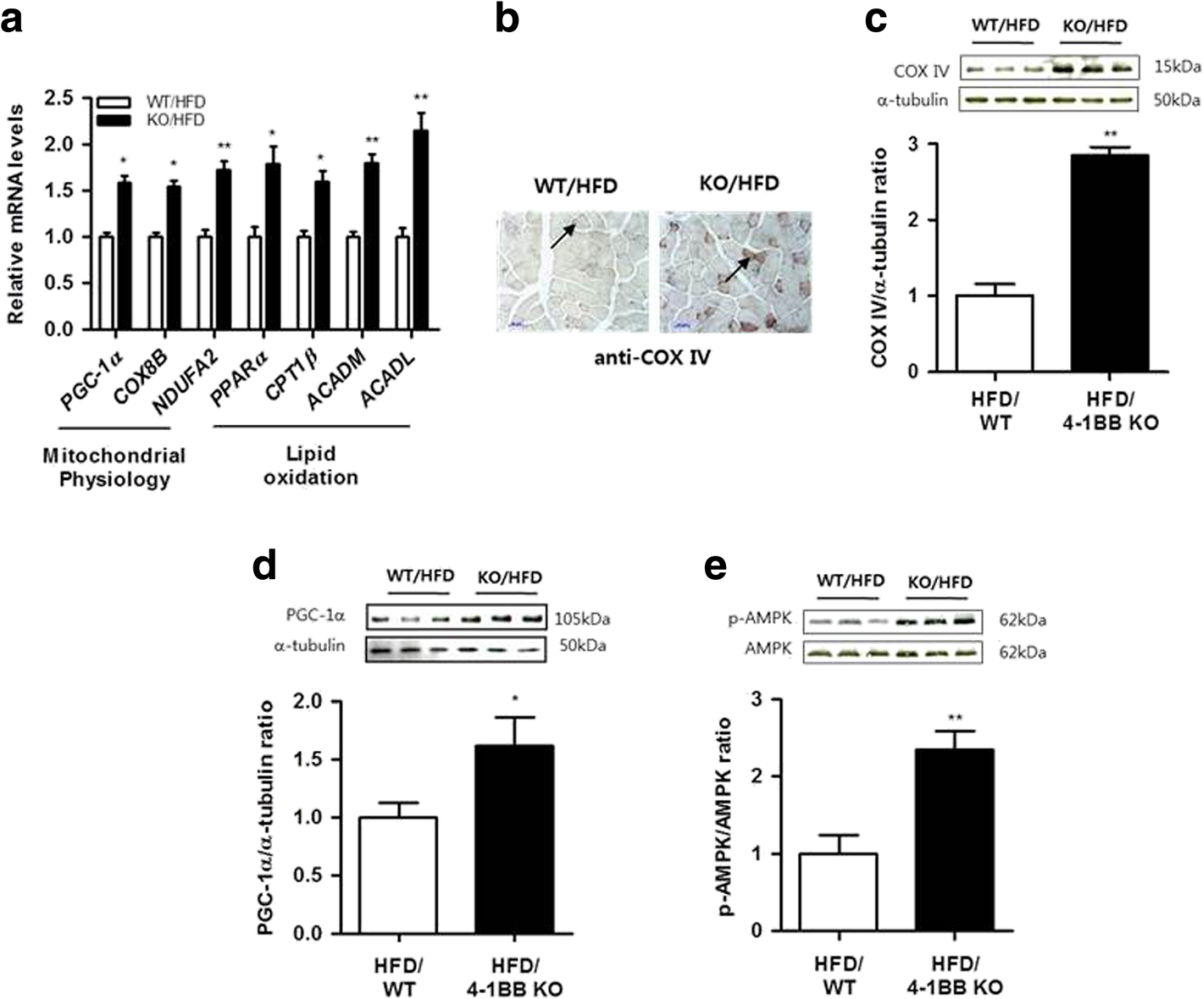
Effect of 4-1BB deficiency on expression of mitochondrial oxidative metabolism-related genes/proteins in the skeletal muscle of HFD-fed obese mice **a** qRT-PCR analysis for markers specific to mitochondrial biogenesis/function (PGC-1α, COX8B, and NDUFA2) and fatty acid oxidation (PPARα, CPT1β, ACADM, and ACADL) in quadriceps muscles. **b** Immunohistochemical staining for COX IV in quadriceps muscle. Arrows indicate stained cells. Magnification × 200; Scale bars 50 μm. **c**-**d** PGC-1α and COX IV protein were measured by Western blotting. The relative intensities of the to α-tubulin control were measured using image J. **e** Phosphorylated AMPK (p-AMPK) and AMPK proteins were measured by Western blotting. The relative intensities of the bands of p-AMPK/AMPK were measured using image J. Data are means ± SEM; n = 6 in each group. *P < 0.05, **P < 0.01 when comparing the WT/HFD and KO/HFD groups

Mitochondrial dysfunction plays a central role in skeletal muscle atrophy [26], and enhancing mitochondrial oxidation is considered to be beneficial for protecting against atrophy. To determine if the protective effect of 4-1BB deficiency against obesity-induced atrophic response in skeletal muscle is associated with oxidative metabolic response, we examined the expression of genes associated with mitochondrial biogenesis and oxidative metabolism in skeletal muscle of WT and 4-1BB-deficient obese mice fed an HFD. Transcript levels of genes involved in regulating mitochondrial biogenesis such as peroxisome proliferator activated receptor gamma coactivator 1 alpha (PGC-1α), as well as those of genes involved in mitochondrial oxidative phosphorylation such as cytochrome c oxidase subunit VIIIB (COX8B) and NADH dehydrogenase ubiquinone 1 alpha subcomplex 2 (NDUFA2), were significantly elevated in the skeletal muscles of HFD-fed 4-1BB-deficient mice. The same results were found for transcripts involved in regulating muscle fatty acid oxidation, including peroxisome proliferator-activated receptor α (PPARα), carnitine palmitoyltransferase 1β (CPT1β), and genes encoding enzymes involved in β-oxidation, including acyl coenzyme A dehydrogenase medium chain (ACADM), and acyl coenzyme A dehydrogenase long chain (ACADL) (Fig. 2a). Consistent with these findings, the muscle from HFD-fed 4-1BB-deficient mice was stained more strongly with antibody against cytochrome-c oxidase subunit IV (COX IV) (Fig. 2b), which is a marker for increased oxidative capacity [27]. Levels of COX IV and PGC-1α protein were also upregulated in the skeletal muscles of HFD-fed 4-1BB-deficient mice (Fig. 2c-d). Moreover, we observed that phosphorylation of AMPK increased in the skeletal muscle of the 4-1BB-deficient obese mice relative to the control (Fig. 2e).

#### Oxidative fiber type in skeletal muscle

**Fig. 3:**
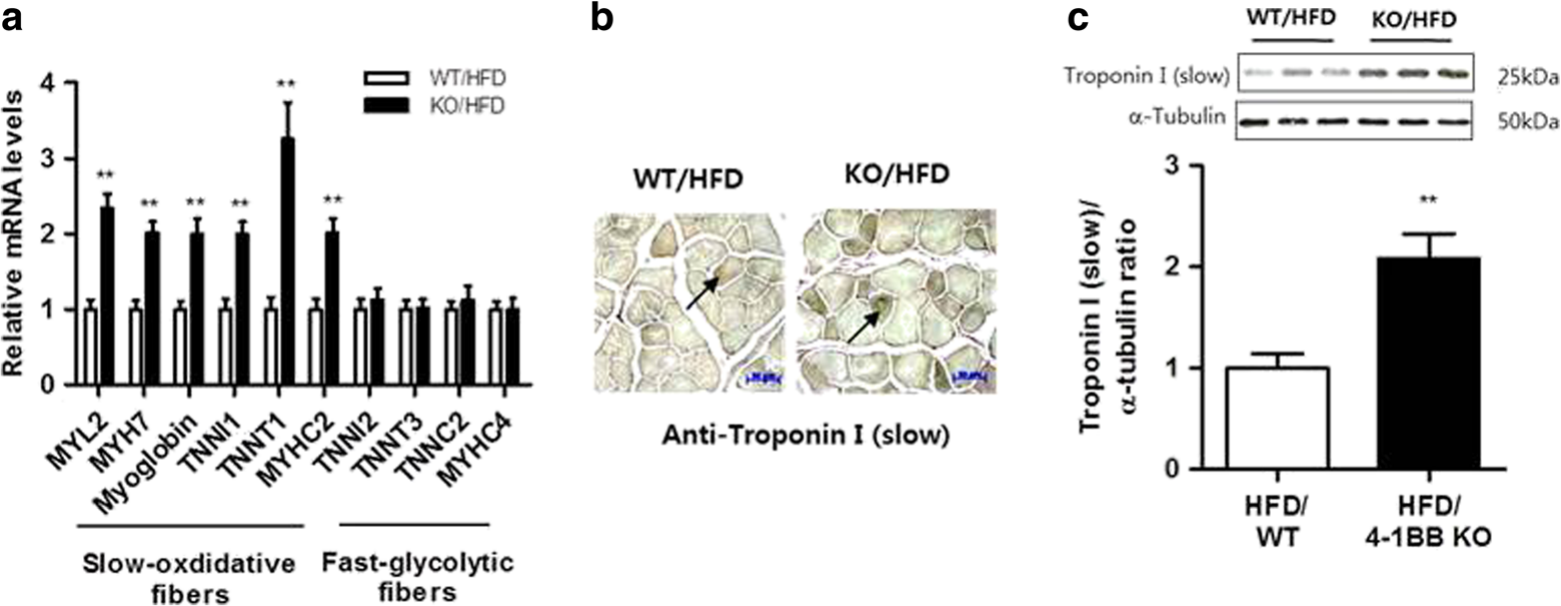
Effect of 4-1BB deficiency on expression of fiber type-related markers in the skeletal muscle of HFD-fed obese mice **a** qRT-PCR analysis for markers specific to slow-oxidative fibers (MYL2, MYH7, myoglobin, TNNI1, TNNT1, and MYHC2) and fast-glycolytic fibers (TNNI2, TNNT3, TNNC2, and MYHC4) in quadriceps muscles. **b** Immunohistochemical staining for troponin I (slow) proteins in quadriceps muscle. Arrows indicate stained cells. Magnification × 200; Scale bars, 50 μm. **c** troponin I (slow) and α-tubulin protein were measured by Western blotting. The relative intensities of the bands of troponin I (slow)/α-tubulin were measured using image J. Data are the means ± SEM; *n* = 6 in each group. **P < 0.01 when comparing the WT/HFD and KO/HFD groups

Muscle remodeling toward an oxidative phenotype, which occurs during physical exercise, protects muscle atrophy [28, 29]. To determine if the metabolic alterations in the skeletal muscle of 4-1BB-deficient obese mice were associated with muscle fiber type, we examined the transcript levels of key genes involved in skeletal muscle fiber types. Indeed, we found that expression of genes specific to type I fibers such as myosin light chain 2 (MYL2), myosin heavy chain 7 (MYH7), myoglobin, troponin I type 1 (TNNI1), troponin T type 1 (TNNT1), as well as those specific to oxidative type IIA fibers such as myosin heavy chain 2 (MYHC2), was greatly elevated in the muscle of the HFD-fed 4-1BB-deficient mice (Fig. 3a), whereas the expression of genes specific to fast, glycolytic type II fibers, such as troponin I type 2 (TNNI2), troponin T type 3 (TNNT3), troponin C type 2 (TNNC2), and myosin heavy chain 4 (MYHC4), did not differ between the groups (Fig. 3a). In agreement with this, histological analysis and western blot analysis showed that HFD-fed 4-1BB-deficient mice contained more Troponin I-staining fibers than HFD-fed WT mice (Fig. 3b-c).

## Discussion

Obesity-induced inflammation is postulated to be a major contributor to skeletal muscle atrophy and metabolic dysregulation. Interestingly, recent studies have shown that the inflammatory molecule TWEAK-Fin14, a member of TNF-TNFR superfamily, plays a crucial role in mediating to skeletal muscle atrophy and metabolic dysfunction [9]. In this study, we used 4-1BB-deficient obese mice fed an HFD to investigate whether the 4-1BBL and 4-1BB system, another member of the TNF-TNFR superfamily, participates in obesity-induced skeletal muscle atrophy. We found that expression of atrophic markers such as MuRF1 and Atrogin-1 was markedly reduced in 4-1BB-deficient obese mice compared with WT control obese mice, and that this was accompanied by an increase in muscle mass as estimated by the tissue weights. Our previous studies showed upregulation of 4-1BB and its ligand in inflamed adipose tissue/skeletal muscle with infiltrated macrophages in obese condition [15, 16], and the interaction of the receptor/ligand augmented obesity-induced production of inflammatory cytokines (TNFα, IL-6, and MCP-1), which was reduced in the absence of 4-1BB [3, 15]. More importantly, we observed that the absence of a 4-1BB-mediated signal suppressed activation of the NF-κB pathway in obese skeletal muscle. These findings indicate that the absence of 4-1BB signal suppresses the NF-κB-mediated inflammatory pathway, and that this is accompanied by increased inflammatory cytokines, which are critical for the ubiquitin-proteasome pathway. Accordingly, the absence of this signal may protect against obesity-induced atrophic response in skeletal muscle. Additionally, given that 4-1BB deficiency lowers the body weight gain and adiposity in HFD-fed obese [16], the improvement of skeletal muscle atrophy in the 4-1BB-deficient obese mice may, at least in part, be associated with the reduction of lipid accumulation.

Several inflammatory receptors/ligands pathways, including Fn14-TWEAK and 4-1BBL-4-1BB, have been shown to activate inflammatory signaling molecules (JNK, NF-κB, ERK, and p38 MAPK) [3, 9, 15, 30]. Activation of these inflammatory signaling molecules is known to reduces the activity of key regulators of oxidative metabolism such as AMPK, PGC-1α and PPARs [31–33], as well as to cause defects in fatty acid oxidation and insulin sensitivity in skeletal muscle [31–33], supporting the link between muscle inflammation and oxidative metabolism. Moreover, inflammation-mediated mitochondria dysfunction accompanied by reduction of oxidative capacity leads to activation of the proteolytic system via the activation of NF-κB [11]. AMPK is a critical regulator of skeletal muscle oxidative metabolism that modulates the expression of transcriptional regulators such as PGC-1α, which is involved in controlling the expression of metabolic and mitochondrial genes [34, 35]. In this study, we investigated whether the increase of mitochondrial genes reflects the reduced atrophic response observed in 4-1BB-deficient obese skeletal muscle. We found that the absence of 4-1BB-mediated inflammatory signaling increased activation of AMPK and transcript levels of the oxidative genes or proteins (COX IV) in the skeletal muscle, indicating that the absence of 4-1BB signal may attenuate obesity-induced muscle oxidative metabolic dysfunction through activation of the AMPK-PGC-1α pathway, leading to protection of atrophic response. Additionally, given the close connection between TNFα and reduced oxidative metabolism (TNFα inhibits AMP-activated protein kinase, leading to the reduction of oxidative capacity in skeletal muscle [36, 37]), the increase of muscle mitochondrial oxidative metabolic genes in 4-1BB-deficient obese mice may be, at least in part, due to a reduction in the level of TNFα in obese skeletal muscle [25].

Skeletal muscle atrophy and/or the derangement of muscle oxidative metabolism are accompanied by changes in the ratio of muscle fiber types [36–38]. It is likely that obese individuals have fewer slow-twitch muscle fibers and more fast-twitch muscle fibers [38]. Moreover, increases in slow-twitch oxidative muscle fibers are thought to protect against intramuscular fat accumulation, insulin resistance [38–40], and muscle atrophy [41]. NF-κB activation is known to serve a dual function by inducing both fast-twitch fiber atrophy and slow-twitch fiber degeneration, while PGC-1α is known to protect slow-twitch oxidative fibers from denervation/immobilization-induced muscle atrophies [42, 43]. The inflammatory cytokine TNFα, a structural homologue of the 4-1BB ligand, is also known to directly suppress PGC-1α in skeletal muscle [33]. In this study, we found that the absence of a 4-1BB signal rescued PGC-1α level in the skeletal muscle of 4-1BB-deficient obese mice, leading to a reduction in the muscle atrophy that occurs under obese conditions. Additionally, 4-1BB deficiency resulted in a great increase in the expression of molecules specific to type I and type IIA muscle fibers, which are rich in mitochondria and have high oxidative capacities [44]. Taken together, these findings suggest that PGC-1α may be an important mechanism by which the absence of 4-1BB signal reduces obesity-induced atrophy and restores mitochondrial oxidative metabolic capacity with oxidative fiber type.

In conclusion, we demonstrated that the absence of 4-1BB protects against obesity-induced muscle atrophy through suppression of NF-κB activation, and that this was associated with restored mitochondrial oxidative metabolic genes with increased slow-twitch fiber-type in the muscle. 4-1BB may be a potential target in combating obesity-related skeletal muscle atrophy and oxidative metabolic dysfunction.

## Abbreviations

ACADL: Acyl coenzyme A dehydrogenase long chain
ACADM: Acyl coenzyme A dehydrogenase medium chain
AMPK: Adenosine monophosphate-activated protein kinase
COX IV: Cytochrome-c oxidase subunit IV
COX8B: Cytochrome c oxidase subunit VIIIB
CPT1β: Carnitine palmitoyltransferase 1 beta
Fn14: Fibroblast growth factor inducible 14
FoxO3: Forkhead box O3
HFD: High-fat diet
IL-6: Interleukin-6
MCP-1: Monocyte chemoattractant protein-1
MuRF1: Muscle RING finger 1
MYH7: Myosin heavy chain 7
MYHC2: Myosin heavy chain 2
MYHC4: Myosin heavy chain 4
MYL2: Myosin light chain 2
NDUFA2: NADH dehydrogenase ubiquinone 1 alpha subcomplex 2
NF-κB: Nuclear factor-kappa B
PGC-1α: Peroxisome proliferator-activated receptor gamma coactivator-1 alpha
PPARα: Peroxisome proliferator activated receptor alpha
TNFα: Tumor necrosis factor alpha
TNNC2: Troponin C type 2
TNNI1: Troponin I skeletal slow type 1
TNNI2: Troponin I type 2
TNNT1: Troponin T skeletal slow type 1
TNNT3: Troponin T type 3
TWEAK: Tumor necrosis factor-like weak inducer of apoptosis
WT: Wild type.

## References

[1] Stenholm S, Harris TB, Rantanen T, Visser M, Kritchevsky SB, Ferrucci L (2008). Sarcopenic obesity: definition, cause and consequences. Curr Opin Clin Nutr Metab Care.

[2] Pellegrinelli V, Rouault C, Rodriguez-Cuenca S, Albert V, Edom-Vovard F, Vidal-Puig A (2015). Human Adipocytes Induce Inflammation and Atrophy in Muscle Cells During Obesity. Diabetes.

[3] Le NH, Kim CS, Tu TH, Choi HS, Kim BS, Kawada T (2013). Blockade of 4-1BB and 4-1BBL interaction reduces obesity-induced skeletal muscle inflammation. Mediators Inflamm.

[4] Varma V, Yao-Borengasser A, Rasouli N, Nolen GT, Phanavanh B, Starks T (2009). Muscle inflammatory response and insulin resistance: synergistic interaction between macrophages and fatty acids leads to impaired insulin action. Am J Physiol Endocrinol Metab.

[5] Lim JP, Leung BP, Ding YY, Tay L, Ismail NH, Yeo A (2015). Monocyte chemoattractant protein-1: a proinflammatory cytokine elevated in sarcopenic obesity. Clin Interv Aging.

[6] Sandri M (2013). Protein breakdown in muscle wasting: role of autophagy-lysosome and ubiquitin-proteasome. Int J Biochem Cell Biol.

[7] Bonaldo P, Sandri M (2013). Cellular and molecular mechanisms of muscle atrophy. Dis Model Mech.

[8] Li H, Malhotra S, Kumar A (2008). Nuclear factor-kappa B signaling in skeletal muscle atrophy. J Mol Med (Berl).

[9] Sato S, Ogura Y, Kumar A (2014). TWEAK/Fn14 Signaling Axis Mediates Skeletal Muscle Atrophy and Metabolic Dysfunction. Front Immunol.

[10] Hindi SM, Mishra V, Bhatnagar S, Tajrishi MM, Ogura Y, Yan Z (2014). Regulatory circuitry of TWEAK-Fn14 system and PGC-1alpha in skeletal muscle atrophy program. FASEB J.

[11] Kumar A, Bhatnagar S, Paul PK (2012). TWEAK and TRAF6 regulate skeletal muscle atrophy. Curr Opin Clin Nutr Metab Care.

[12] Wang Y, Pessin JE (2013). Mechanisms for fiber-type specificity of skeletal muscle atrophy. Curr Opin Clin Nutr Metab Care.

[13] Hurtado J, Kim S, Pollok K, Lee Z, Kwon B (1995). Potential role of 4-1BB in T cell activation. Comparison with the costimulatory molecule CD28. J Immunol.

[14] Olofsson PS, Söderström LÅ, Wågsäter D, Sheikine Y, Ocaya P, Lang F (2008). CD137 Is Expressed in Human Atherosclerosis and Promotes Development of Plaque Inflammation in Hypercholesterolemic Mice. Circulation.

[15] Tu TH, Kim CS, Goto T, Kawada T, Kim BS, Yu R. 4-1BB/4-1BBL Interaction Promotes Obesity-Induced Adipose Inflammation by Triggering Bidirectional Inflammatory Signaling in Adipocytes/Macrophages. Mediat Inflamm. 2012;2012:972629. 10.1155/2012/972629 PMC3534384 23316108

[16] Kim C-S, Kim JG, Lee B-J, Choi M-S, Choi H-S, Kawada T (2011). Deficiency for Costimulatory Receptor 4-1BB Protects Against Obesity-Induced Inflammation and Metabolic Disorders. Diabetes.

[17] Vinay DS, Kwon BS (1998). Role of 4-1BB in immune responses. Semin Immunol.

[18] Takahashi C, Mittler RS, Vella AT (1999). Cutting Edge: 4-1BB Is a Bona Fide CD8 T Cell Survival Signal. J Immunol.

[19] Tu TH, Kim CS, Nam-Goong IS, Nam CW, Kim YI, Goto T (2015). 4-1BBL signaling promotes cell proliferation through reprogramming of glucose metabolism in monocytes/macrophages. FEBS J.

[20] Kwon BS, Hurtado JC, Lee ZH, Kwack KB, Seo SK, Choi BK (2002). Immune Responses in 4-1BB (CD137)-Deficient Mice. J Immunol.

[21] Ju TJ, Kwon WY, Kim YW, Kim JY, Kim YD, Lee IK (2014). Hemin improves insulin sensitivity in skeletal muscle in high fat-fed mice. J Pharmacol Sci.

[22] Shintaku J, Peterson JM, Talbert EE, Gu JM, Ladner KJ, Williams DR (2016). MyoD Regulates Skeletal Muscle Oxidative Metabolism Cooperatively with Alternative NF-kappaB. Cell Rep.

[23] Jackman RW, Kandarian SC (2004). The molecular basis of skeletal muscle atrophy. Am J Physiol Cell Physiol.

[24] Glass DJ (2005). Skeletal muscle hypertrophy and atrophy signaling pathways. Int J Biochem Cell Biol.

[25] Le NH, Kim CS, Park T, Park JH, Sung MK, Lee DG (2014). Quercetin protects against obesity-induced skeletal muscle inflammation and atrophy. Mediators Inflamm.

[26] Liu J, Peng Y, Wang X, Fan Y, Qin C, Shi L (2016). Mitochondrial Dysfunction Launches Dexamethasone-Induced Skeletal Muscle Atrophy via AMPK/FOXO3 Signaling. Mol Pharm.

[27] Fontanesi F, Soto IC, Horn D, Barrientos A (2006). Assembly of mitochondrial cytochrome c-oxidase, a complicated and highly regulated cellular process. Am J Physiol Cell Physiol.

[28] Lanza IR, Short DK, Short KR, Raghavakaimal S, Basu R, Joyner MJ (2008). Endurance exercise as a countermeasure for aging. Diabetes.

[29] Egan B, Zierath JR (2013). Exercise metabolism and the molecular regulation of skeletal muscle adaptation. Cell Metab.

[30] Dempsey PW, Doyle SE, He JQ, Cheng G (2003). The signaling adaptors and pathways activated by TNF superfamily. Cytokine Growth Factor Rev.

[31] Steinberg GR, Michell BJ, van Denderen BJ, Watt MJ, Carey AL, Fam BC (2006). Tumor necrosis factor alpha-induced skeletal muscle insulin resistance involves suppression of AMP-kinase signaling. Cell Metab.

[32] Gelinas DS, McLaurin J (2005). PPAR-alpha expression inversely correlates with inflammatory cytokines IL-1beta and TNF-alpha in aging rats. Neurochem Res.

[33] Palomer X, Alvarez-Guardia D, Rodriguez-Calvo R, Coll T, Laguna JC, Davidson MM (2009). TNF-alpha reduces PGC-1alpha expression through NF-kappaB and p38 MAPK leading to increased glucose oxidation in a human cardiac cell model. Cardiovasc Res.

[34] Handschin C, Spiegelman BM (2006). Peroxisome proliferator-activated receptor gamma coactivator 1 coactivators, energy homeostasis, and metabolism. Endocr Rev.

[35] McGee SL, Hargreaves M (2010). AMPK-mediated regulation of transcription in skeletal muscle. Clin Sci (Lond).

[36] Yan Z, Okutsu M, Akhtar YN, Lira VA (2011). Regulation of exercise-induced fiber type transformation, mitochondrial biogenesis, and angiogenesis in skeletal muscle. J Appl Physiol.

[37] Tang K, Wagner PD, Breen EC (2010). TNF-α-mediated reduction in PGC-1α may impair skeletal muscle function after cigarette smoke exposure. J Cell Physiol.

[38] Tanner CJ, Barakat HA, Dohm GL, Pories WJ, MacDonald KG, Cunningham PRG (2002). Muscle fiber type is associated with obesity and weight loss. Am J Physiol Endocrinol Metab.

[39] Kelley DE, Goodpaster B, Wing RR, Simoneau JA (1999). Skeletal muscle fatty acid metabolism in association with insulin resistance, obesity, and weight loss. Am J Physiol.

[40] Consitt LA, Bell JA, Houmard JA (2009). Intramuscular lipid metabolism, insulin action, and obesity. IUBMB Life.

[41] Henique C, Mansouri A, Fumey G, Lenoir V, Girard J, Bouillaud F (2010). Increased mitochondrial fatty acid oxidation is sufficient to protect skeletal muscle cells from palmitate-induced apoptosis. J Biol Chem.

[42] Wenz T, Rossi SG, Rotundo RL, Spiegelman BM, Moraes CT (2009). Increased muscle PGC-1alpha expression protects from sarcopenia and metabolic disease during aging. Proc Natl Acad Sci U S A.

[43] Brault JJ, Jespersen JG, Goldberg AL (2010). Peroxisome proliferator-activated receptor gamma coactivator 1alpha or 1beta overexpression inhibits muscle protein degradation, induction of ubiquitin ligases, and disuse atrophy. J Biol Chem.

[44] Zierath JR, Hawley JA (2004). Skeletal Muscle Fiber Type: Influence on Contractile and Metabolic Properties. PLoS Biol.

